# Clinical Heterogeneity of Major Depressive Disorder: The Role of Trauma, Dissociation, and Sleep

**DOI:** 10.3390/jcm15062364

**Published:** 2026-03-19

**Authors:** Zeynep Namlı, Lut Tamam, Mehmet Emin Demirkol, Mahmut Onur Karaytuğ, Caner Yeşiloğlu, Sinem Çetin Demirtaş, Kerim Uğur

**Affiliations:** 1 Department of Psychiatry, Faculty of Medicine, Cukurova University, Adana 01330, Turkey; ltamam@gmail.com (L.T.); emindemirkol@gmail.com (M.E.D.); mokaraytug@gmail.com (M.O.K.); yesiloglucaner@gmail.com (C.Y.); cetnsinem@gmail.com (S.Ç.D.); 2 Department of Psychiatry, Malatya Turgut Özal University; Malatya 44900, Turkey; premirek@gmail.com

**Keywords:** childhood trauma, dissociation, major depressive disorder, posttraumatic growth, suicide, sleep quality

## Abstract

**Background:** Major depressive disorder (MDD) is a common mental disorder characterized by a wide range of symptoms and a substantial contribution to global disease burden. Our study aimed to examine the relationships between childhood trauma, sleep quality, dissociative symptoms, posttraumatic growth, and suicidality in patients diagnosed with MDD. **Methods:** Our sample consisted of 115 patients with MDD and 84 healthy controls. Participants were administered the Hamilton Depression Rating Scale (HDRS), Beck Scale for Suicidal Ideation (BSSI), Pittsburgh Sleep Quality Index (PSQI), Childhood Trauma Questionnaire (CTQ-33), Dissociative Experiences Scale (DES), and Posttraumatic Growth Inventory (PTGI). Group differences were analyzed, and associations among variables within the MDD group were examined using correlation and mediation analyses. **Results:** Compared to healthy controls, patients with MDD had significantly higher scores on all scales except the PTGI (*p* < 0.001 for each). Within the MDD group, individuals with a history of suicide attempts had significantly higher CTQ total scores, physical and sexual abuse subscale scores, and DES scores than those without previous attempts. Additionally, dissociative experiences had a partial mediating role in the relationship between depression severity and suicidal ideation, as well as in the relationship between childhood traumas and sleep quality. **Conclusions:** The findings highlight the clinical relevance of dissociative experiences and sleep disturbances in the heterogeneous presentation of MDD and their association with illness severity and suicidality. In the follow-up and treatment process of patients with MDD, risk and protective factors should be evaluated together, and individualized treatment programs should be targeted.

## 1. Introduction

Major depressive disorder (MDD) is one of the most prevalent psychiatric disorders worldwide. According to the World Health Organization, depression affects approximately 5.7% of adults globally, and its prevalence continues to increase [1]. As a leading cause of disability [2], MDD is strongly associated with suicidal phenomena, with suicidal ideation and attempts reported in nearly one-third to one-half of affected individuals [3].

Sleep disturbances are highly prevalent among individuals with depression. Insomnia, in particular, constitutes one of the core symptoms of depression and has also been recognized as a risk factor that increases vulnerability to depressive disorders [4]. Several mechanisms have been proposed to explain this association. Genetic studies have demonstrated a significant overlap between insomnia and MDD, suggesting a shared biological vulnerability [5]. Furthermore, disturbances in circadian rhythms may heighten vulnerability to depressive symptoms [4], whereas sleep disturbances may induce inflammatory processes associated with the onset and progression of depression [6]. MDD accompanied by insomnia has been associated with more severe depressive manifestations, poorer response to antidepressant treatment, and impaired social functioning [7]. Moreover, severe insomnia in individuals with depressive disorders has been identified as a clinical predictor of suicidal behavior [8].

Childhood trauma (CT), including sexual, physical, and emotional abuse as well as physical and emotional neglect, is a well-established risk factor for the development of MDD [9]. Evidence suggests that these subtypes may differentially influence the clinical presentation and course of depression. Emotional abuse and neglect have been strongly associated with greater depressive symptom severity, cognitive vulnerability, and persistent negative cognitive schemas [10]. Physical abuse has been linked to impulsivity, emotion regulation difficulties, and heightened stress reactivity, which may contribute to more severe depressive episodes and increased suicidality [11]. Sexual abuse has been associated with earlier onset of depression, higher psychiatric comorbidity, and poorer treatment outcomes [12]. Childhood neglect has also been related to impairments in interpersonal functioning and attachment patterns that may predispose individuals to recurrent depressive episodes [13]. Overall, individuals with a history of CT tend to show earlier onset of MDD, more severe symptoms, poorer treatment response, and an increased risk of suicidal ideation and behavior.

Numerous studies have demonstrated that individuals exposed to early life adversity are more likely to develop dissociative symptoms later in life [14]. Dissociation is commonly conceptualized as a psychological response to excessive stress that allows individuals to detach from distressing internal experiences [15]. Although it may initially serve as an adaptive coping mechanism, persistent dissociation can disrupt emotional processing, memory integration, and the development of a coherent sense of self [15,16]. These disruptions may impair emotion regulation and interpersonal functioning, increasing vulnerability to depressive symptoms. In individuals with a history of CT, dissociation may therefore represent a psychological pathway linking traumatic experiences to depression and suicidal ideation [14,17]. Moreover, the co-occurrence of depression and dissociation has been related to higher suicide risk, poorer psychosocial functioning, and greater treatment resistance [18].

In contrast to the adverse outcomes following traumatic experiences, positive changes have also long been recognized by researchers. A substantial proportion of trauma survivors are able to heal, and some may even experience favorable personal transformation in the form of posttraumatic growth (PTG) as a result of traumatic conditions [19]. Such positive changes may occur in different domains, including self-perception, relationships with others, and life philosophy. Individuals who overcome trauma may begin to view themselves as stronger and more capable of coping with difficult events. Their perception of others may change, leading to a greater sense of closeness, and they may also develop a stronger sense of purpose and new life priorities [20]. Although psychopathology and positive functioning are often assumed to lie at opposite ends of a spectrum, evidence indicates that the positive and negative consequences of trauma—such as mental disorder and PTG symptoms—can be experienced simultaneously [21].

MDD is clinically heterogeneous, with considerable variability in symptoms, etiology, and course across individuals [22]. Researchers have widely investigated trauma-related factors—such as CT, dissociative symptoms, and suicidality—in relation to MDD, as these may contribute to its heterogeneity. In contrast, the potential role of PTG in MDD has received little attention. Understanding how PTG relates to CT and other clinical variables may provide new insights into the clinical heterogeneity of depression and offer new perspectives on treatment strategies. Our study, therefore, aimed to examine the relationships among CT, sleep quality, dissociative symptoms, PTG, and suicidality in individuals diagnosed with MDD.

## 2. Method

### 2.1. Sample and Procedure

The study was conducted in the outpatient clinics of the Department of Psychiatry at Çukurova University Faculty of Medicine, between April 2023 and April 2024. The sample consisted of two groups: patients diagnosed with MDD and healthy volunteers without any psychiatric disorder, matched for age and sex distribution. To ensure that participants could reliably complete the self-report scales and provide accurate reports of past experiences, literate individuals aged 18 to 40 were included in the study. This age range was selected to limit the potential impact of long-term memory distortions, as the subjective impact and interpretation of traumatic experiences may change over time. Before participation, all individuals were informed about the study and provided written informed consent.

In the first stage, the first author conducted a psychiatric interview based on the diagnostic criteria of the Diagnostic and Statistical Manual of Mental Disorders, Fifth Edition (DSM-5) [23]. Following diagnostic confirmation, the data form, Hamilton Depression Rating Scale (HDRS), and Beck Scale for Suicidal Ideation (BSSI) were administered. In the MDD group, individuals with severe depression (HDRS ≥ 24) [24], psychotic symptoms, comorbid psychiatric disorders, or alcohol/substance use disorders were excluded from the study. Additionally, individuals who had received electroconvulsive therapy within the past three months were excluded due to potential cognitive side effects [25]. Only participants receiving antidepressant treatment were included. In the control group, individuals with HDRS ≤ 7 [24], no lifetime history of psychiatric disorders or suicide attempts, and no use of psychiatric medication were included.

In the second stage, participants completed the Childhood Trauma Questionnaire (CTQ-33), the Dissociative Experiences Scale (DES), the Pittsburgh Sleep Quality Index (PSQI), and the Posttraumatic Growth Inventory (PTGI). To minimize the impact of environmental stimuli, the clinician conducted the interviews face-to-face in a separate room. Approximately 40–50 min was allocated for each participant, and any points that participants did not understand were clarified.

Seven patients with MDD and three healthy controls who withdrew from the study or completed the scales incompletely were excluded. The final sample consisted of 115 patients with MDD and 84 healthy controls. A post hoc power analysis was conducted using G*Power 3.1.9.2 to assess whether the study had sufficient statistical power [26]. For two-group comparisons, with group sizes of 115 and 84 participants, an effect size of 0.5, a significance level of 0.05, and two-tailed analyses, the achieved power was 0.93 (93%). These results confirm that the sample size was sufficient.

The study was approved by the Non-Interventional Clinical Research Ethics Committee of Çukurova University Faculty of Medicine on 4 November 2022 at meeting no. 127 (decision no: 74).

### 2.2. Measures

#### 2.2.1. Data Form

The first section of the data form assessed participants’ age, sex, marital status, occupation, place of residence, history of physical illness, and smoking habits. The second section evaluated the clinical characteristics of patients diagnosed with MDD, including duration of illness, history of hospitalization, history of suicide attempts, and pharmacological treatment. Among individuals with a history of self-harm behavior, all acts carried out with the intention of ending one’s life were considered suicide attempts.

#### 2.2.2. The Hamilton Depression Rating Scale (HDRS)

The HDRS, widely used to assess the severity of depression, consists of 17 items and is administered by a clinician. Higher total scores indicate greater severity of depression [27]. Akdemir et al. conducted the Turkish validity and reliability study and reported a Cronbach’s alpha value of 0.75 [28].

#### 2.2.3. The Beck Scale for Suicidal Ideation (BSSI)

The BSSI, which consists of nineteen items and five sections, assesses suicidal ideation, plans, and intent. It is administered by a clinician, and higher total scores indicate greater suicide risk [29]. In the Turkish validity and reliability study, the Cronbach’s alpha value was reported as 0.84 [30].

#### 2.2.4. The Pittsburgh Sleep Quality Index (PSQI)

The PSQI is a self-report questionnaire that assesses sleep quality and sleep disturbances. It consists of nineteen items and includes seven components. The total score is obtained by summing the scores of these seven components, with higher total scores indicating poorer sleep quality [31]. In the Turkish validity and reliability study, the Cronbach’s alpha value was reported as 0.80 [32].

#### 2.2.5. The Childhood Trauma Questionnaire (CTQ-33)

The CTQ is a self-report questionnaire that assesses childhood abuse and neglect. Şar et al. developed the CTQ-33 by adding the overprotection–overcontrol (OP-OC) dimension to the Turkish version of the CTQ-28. The OP-OC dimension includes items such as “My family interfered in everything I did” and “My family restricted my contact with peers and friends.” The questionnaire comprises the subscales of emotional abuse, emotional neglect, physical abuse, physical neglect, sexual abuse, OP-OC, and denial. The total score is calculated by summing the scores of the six subscales, excluding the denial items. Şar et al. reported a Cronbach’s alpha value of 0.87 for the CTQ-33 [33].

#### 2.2.6. The Dissociative Experiences Scale (DES)

The DES is a 28-item self-report questionnaire that assesses dissociative experiences and their severity. Participants rate dissociative symptoms not related to alcohol or substance use on a scale ranging from 0% to 100%. The scale score is calculated as the arithmetic mean of the item scores, with higher scores indicating more severe dissociative symptoms [34]. In the Turkish validity and reliability study, the Cronbach’s alpha value was 0.91 [35].

#### 2.2.7. The Posttraumatic Growth Inventory (PTGI)

The PTGI is a self-report inventory that assesses perceived psychological growth following traumatic experiences. Higher scores indicate positive psychological changes associated with adverse life events [36]. The Turkish version of the scale consists of 21 items. In the validity and reliability study conducted by Kağan et al., the Cronbach’s alpha value was 0.92 [37].

## 3. Statistical Analysis

The distributional properties of continuous variables were evaluated using skewness and kurtosis coefficients (an acceptable range of −1.5 to +1.5), standardized *z*-scores (an acceptable range of −3.29 to +3.29), and a visual inspection of histograms. Variables meeting these criteria were considered to be approximately normally distributed. Continuous variables were expressed as the mean ± standard deviation if they were normally distributed, or as the median (minimum—maximum) if the assumption of normality was violated. Categorical variables were summarized as frequencies and percentages.

Between-group comparisons (MDD versus control group) were conducted using independent samples *t*-tests for normally distributed continuous variables, and Mann–Whitney *U* tests for non-normally distributed variables. The Pearson chi-square test was used for 2 × 2 tables with five or more expected cell counts to compare differences between categorical variables. Cohen’s *d* for parametric and *r* values for non-parametric analyses were calculated to determine the extent of group differences.

Within the MDD group, associations among clinical scale scores were examined using correlation analyses. Pearson’s correlation coefficient was applied to variables demonstrating normal distribution, while Spearman’s *rho* correlation coefficient was used when normality assumptions were not met.

Two separate mediation models were examined within the MDD group using PROCESS Model 4. The first model evaluated the mediating role of dissociative experiences (DES) in the relationship between depression severity (HDRS) and suicidal ideation (BSSI). The second model investigated the mediating role of dissociative experiences in the relationship between childhood trauma (CTQ total score) and sleep quality (PSQI score). In both models, all variables were treated as continuous. Total, direct, and indirect effects were estimated. Indirect effects were tested using a bootstrapping procedure with 5000 resamples, and bias-corrected 95% confidence intervals were calculated. However, given the cross-sectional nature of the data, these results have been interpreted as indicative of statistical mediation rather than causal mediation.

All statistical analyses were conducted using IBM SPSS Statistics version 26.0 (IBM Corporation, Armonk, NY, USA). Mediation analyses were performed using the PROCESS macro (Model 4) [38]. The significance level was set at 0.05 for all analyses, and statistically significant values were bold in the tables.

## 4. Results

Table 1 presents a comparison of the sociodemographic features of the MDD and control groups. There were no statistically significant differences between the two groups in terms of age, gender, marital status, place of residence, or smoking status (*p* > 0.05 for each). Unemployment and a family history of mental disorder were significantly higher in the MDD group (*p* < 0.001).

In the MDD group, the mean duration of disorder was 3.74 ± 3.55 years, the mean number of psychiatric hospitalizations was 0.23 ± 0.84, and the number of hospitalized patients was 27 (23.5%). Thirty-one of the patients (26.9%) had a history of suicide attempts, and 14 of them had multiple attempts. Seven of the patients (6.1%) had a history of electroconvulsive therapy (ECT). All of the MDD group were currently receiving only antidepressant treatment.

The comparison of scale scores revealed that all scores of the patients with MDD were significantly higher than those of the control group, except for the PTGI (*p* < 0.001 for each). The control group had significantly higher PTGI scores than the MDD group (*p* < 0.001). Table 2 presents the participants’ scale scores.

Table 3 presents the correlation analyses of the MDD group’s scale scores. In the MDD group, statistically significant weak-to-moderate positive linear correlations were found between the HDRS score and the BSSI, DES, CTQ total, emotional neglect, physical neglect, physical abuse, and PSQI scores (*p* < 0.05 for each). A statistically significant moderate linear correlation was also observed between the HDRS score and the PTGI score in the inverse direction (*p* < 0.001).

Positive linear correlations were observed between the BSSI score and DES (moderate), sexual abuse (weak), and PSQI (weak-to-moderate) scores (*p* < 0.05 for each). There were statistically significant weak-to-moderate positive linear correlations between the DES score and the CTQ total, emotional neglect, emotional abuse, physical neglect, and physical abuse scores (*p* < 0.05 for each). Additionally, a moderate positive linear correlation was observed between the DES and PSQI scores (*p* < 0.001).

Moderate-to-strong positive linear correlations were observed between the CTQ total score and all CTQ subscale scores (emotional neglect, emotional abuse, physical neglect, physical abuse, sexual abuse, and OP-OC) (*p* < 0.001 for each). There was also a significant linear relationship between the CTQ total and PSQI scores in the same direction (*p* < 0.001).

Finally, statistically significant positive linear correlations were determined between the PSQI score and emotional neglect (moderate), emotional abuse (weak-to-moderate), physical neglect (weak), and physical abuse (weak-to-moderate) scores (*p* < 0.05 for each).

In the MDD group, patients with a history of suicide attempts had significantly higher BSSI and DES scores compared to those without previous attempts (*p* < 0.001 and *p* = 0.042, respectively). In addition, CTQ total scores, as well as physical abuse and sexual abuse subscale scores, were significantly higher in patients with previous suicide attempts (*p* = 0.016, *p* = 0.021, and *p* = 0.011, respectively). No significant differences were observed between suicide attempters and non-attempters in terms of HDRS, PTGI, PSQI, CTQ emotional abuse, emotional neglect, physical neglect, or OP-OC subscale scores (*p* > 0.05 for each). Comparisons between MDD patients with and without previous suicide attempts are presented in Table 4.

### Mediation Analyses

In patients diagnosed with MDD, the mediating role of dissociative experiences in the relationship between depression severity and suicidal ideation was tested. In this analysis, the response variable (*Y*) was the suicidal ideation (BSSI score), the independent variable (*X*) was the depression severity (HDRS score), and the mediator variable (*M*) was the dissociative experiences (DES score).

Depression severity (HDRS score) significantly predicted suicidal ideation (BSSI score) in the model without the mediator (DES score) (*β* = 0.426, *p* < 0.001). After the mediator was included in the model, depression severity significantly predicted dissociative experiences (DES score) (*β* = 1.103, *p* < 0.001), and dissociative experiences significantly predicted suicidal ideation (*β* = 0.108, *p* < 0.001). The direct path from depression severity to suicidal ideation remained significant after the inclusion of the mediator (*β* = 0.306, *p* = 0.005).

**Figure 1:**
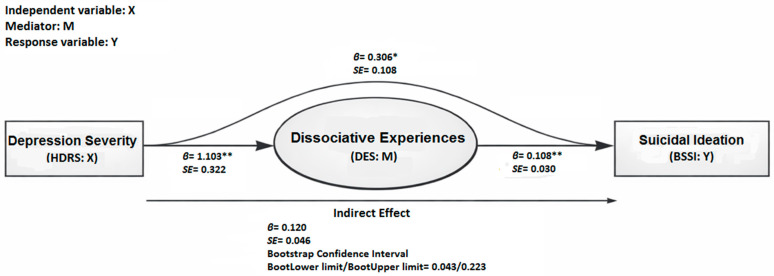
The mediator role of dissociative experiences in the relationship between depression severity and suicidal ideation. HDRS: Hamilton Depression Rating Scale, BSSI: Beck Scale for Suicidal Ideation, DES: Dissociative Experiences Scale; *p* < 0.05 = *, *p* < 0.001 = **.

Figure 1 presents the path diagram with standardized path coefficients for the mediation model including the DES score. Bootstrap analysis indicated that the indirect association between depression severity and suicidal ideation through dissociative experiences was statistically significant, consistent with a partial mediation model. Accordingly, dissociative experiences had a partial mediating role in the association between depression severity and suicidal ideation (Table 5; Figure 1).

Additionally, we tested the mediating role of dissociative experiences in the relationship between childhood traumas and sleep quality in patients diagnosed with MDD. In this analysis, the response variable (*Y*) was the sleep quality (PSQI score), the independent variable (*X*) was the childhood traumas (CTQ total score), and the mediator variable (*M*) was the dissociative experiences (DES score).

Childhood traumas (CTQ total score) significantly predicted sleep quality (PSQI score) in the model without the mediator (DES score) (*β* = 0.066, *p* = 0.001). After the mediator was included in the model, childhood traumas significantly predicted dissociative experiences (DES score) (*β* = 0.347, *p* < 0.001), and dissociative experiences significantly predicted sleep quality (*β* = 0.064, *p* < 0.001). The direct path from childhood traumas to sleep quality remained significant after the inclusion of the mediator (*β* = 0.043, *p* = 0.032).

**Figure 2:**
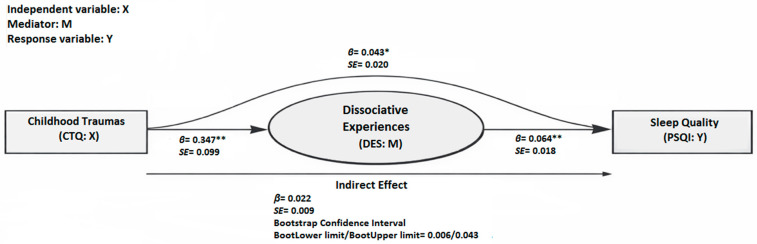
The mediator role of dissociative experiences in the relationship between childhood traumas and sleep quality. DES: Dissociative Experiences Scale, CTQ: Childhood Trauma Questionnaire, PSQI: Pittsburgh Sleep Quality Index; *p* < 0.05 = *, *p* < 0.001 = **.

Figure 2 presents the path diagram with standardized path coefficients for the mediation model including the DES score. Bootstrap analysis indicated that the indirect association between childhood traumas and sleep quality through dissociative experiences was statistically significant, consistent with a partial mediation model. Accordingly, dissociative experiences had a partial mediating role in the association between childhood traumas and sleep quality (Table 6; Figure 2).

## 5. Discussion

The principal finding of our study, in which we concurrently evaluated positive/negative and modifiable/non-modifiable variables, is the significant association between dissociative experiences and both sleep quality and suicide risk in MDD. Our findings further suggest that dissociative processes, which are frequently overlooked, play a substantial role in the clinical presentation of MDD.

CT is a common underlying risk factor for depression and is associated with the heterogeneous nature of the disorder [39]. Gardner et al. reported that individuals exposed to sexual and/or physical abuse in childhood had a twofold increased risk of depression [40]. In our study, CT was reported more frequently among patients with MDD than among healthy controls and was associated with more severe depressive symptoms, dissociative experiences, and poorer sleep quality. Our findings support the notion that CT is associated not only with increased vulnerability to MDD in adulthood but also with greater illness severity and greater symptom diversity [41,42].

There is robust evidence that CT increases the risk of suicidal ideation and behaviors [43]. It has been reported that the likelihood of experiencing suicidal ideation and/or attempting suicide in adulthood is 3.5 times higher among individuals who were sexually abused in childhood, and approximately two times higher among those who experienced physical abuse [44]. Similarly, our study revealed that, among patients with MDD, a history of sexual abuse was associated with suicidal ideation, while histories of physical and sexual abuse and greater trauma severity were associated with lifetime suicide attempts.

The risk of suicidal ideation and attempts increases with the number of adverse experiences faced during childhood. Notably, the emergence of psychiatric disorders cannot solely explain the impact of CT on suicidal behavior in adulthood [45]. Risk factors, including CT, directly affect the pathophysiological mechanisms of MDD, frequently interacting in a complex, non-linear process. CT may trigger negative beliefs and maladaptive behaviors, which can contribute to increased suicide risk [46]. Since the predictive capacity of single risk factors for future suicidal ideation and behaviors is inherently limited, it has been recommended that multiple risk factors be examined simultaneously [18]. Accordingly, in our study, in addition to CT, dissociative experiences and poor sleep quality were also associated with suicide risk among patients with MDD.

Dissociative symptoms, which are not included among the classical dimensions associated with MDD in diagnostic systems, appear to contribute to depressive psychopathology [47]. Dissociation has been reported not only by individuals with depressive disorders but also by members of the general population experiencing depressive symptoms [48]. In our study, dissociative experiences were more prevalent in the MDD group and were positively associated with symptom severity. These findings can be interpreted bidirectionally: increased depression severity may trigger dissociative symptoms, or dissociative experiences may worsen the clinical course of the disorder.

According to trauma models, dissociative experiences serve as primary defense mechanisms for coping with overwhelming and stressful experiences, particularly in situations where escape is not possible [15,49]. Meta-analytic data indicate that individuals reporting higher levels of childhood maltreatment also report higher levels of dissociation in adulthood [50]. In our study, the association between CT severity and dissociative experiences may be explained by dissociation’s role in distancing individuals from distressing emotions linked to traumatic events [48].

Research on dissociation as a suicide risk factor suggests that dissociation may mediate the relationship between depression and self-harm behaviors [51]. In our study, dissociative experiences were significantly associated with suicidality, and dissociation partially mediated the relationship between depression severity and suicidal ideation. Dissociative symptoms appear to reflect attempts to disconnect from distressing thoughts and emotions [47]. Similarly, suicidal behavior can be understood as an extreme coping strategy aimed at escaping unbearable internal experiences [52]. Although dissociation and suicidal behavior are phenomenologically distinct, both may serve the function of distancing the individual from intolerable psychological states. Therefore, in depressive disorders, the presence of dissociative symptoms should be considered not only as a consequence of trauma but also as a clinically relevant indicator of suicide risk.

Longitudinal studies indicate a bidirectional relationship between sleep disturbances and depression: poor sleep quality predicts subsequent depressive symptoms, while depressive disorders contribute to various sleep problems [53]. Consistent with this evidence, our findings demonstrated that sleep quality was significantly poorer in patients with MDD than in healthy controls and that it was associated with the severity of depression. Therefore, addressing sleep disturbances in patients with MDD could potentially reduce the severity of depressive episodes.

Given the high prevalence of sleep disorders in various populations, sleep quality has emerged as a key area of research interest [54]. In our study, sleep quality was significantly associated not only with depression severity but also with suicidal ideation, dissociative experiences, and CT. These results suggest that sleep disturbances in MDD may be more than just a symptom of depression, potentially being linked to multiple psychopathological dimensions.

Sleep problems negatively affect daily functioning, as well as behavioral and emotional processes. Previous research suggests that impaired sleep quality may increase suicidal ideation by heightening emotional reactivity and intensifying negative affective processes [55]. Consistent with this literature, our findings indicate that poorer sleep quality is associated with increased suicidal ideation in individuals with MDD. Identifying dynamic and modifiable risk factors, such as sleep quality, may enhance our understanding of periods of heightened suicide risk [56] and inform targeted suicide prevention strategies.

Numerous studies have demonstrated an association between poor sleep quality, various sleep disturbances, and CT [57,58]. Although dissociation is a common symptom among individuals with a history of trauma, its relationship with sleep disturbances has been examined in relatively fewer studies. Consistent with previous findings [57,59], our study revealed that dissociative experiences were associated with poorer sleep quality in patients with MDD.

Moreover, our results indicated that dissociative experiences were related to the association between CT and sleep quality, supporting a partial mediating role. Dissociation is a transdiagnostic concept and is associated with poor treatment outcomes [60]. Understanding how dissociative processes interact with sleep disturbances and other clinical variables can guide more individualized treatment strategies. Targeted interventions addressing dissociative symptoms—such as phase-oriented therapy, individual psychotherapy, and group therapy—may therefore improve clinical outcomes and reduce suicide risk in patients with MDD [61]. Furthermore, these findings may provide indirect support for the proposed subtype of “dissociative depression” [62] and underscore the need for further research in this area.

Protective factors enhance an individual’s ability to maintain or regain mental health when faced with adversity. Accordingly, recent research has not only focused on risk factors, but also on protective constructs such as resilience and PTG. PTG is defined as positive psychological change that emerges following traumatic experiences, involving the disruption of core beliefs and subsequent cognitive restructuring processes [63]. In contrast, depression is characterized by feelings of hopelessness and worthlessness, cognitive rigidity, and negative automatic thoughts. These cognitive patterns may hinder the meaning-making and cognitive reframing processes necessary for PTG development. Consistent with this, our findings indicated that PTG levels were lower in the MDD group than in healthy controls, and PTG was inversely associated with depression severity. These findings suggest that as the severity of depressive symptoms increases, an individual’s capacity to process traumatic experiences may decrease.

Higher levels of PTG have been associated with lower suicidal ideation in both military [64] and community samples [65]. However, in our study, PTG was not significantly associated with suicidal ideation or previous suicide attempts among patients with MDD. This discrepancy may be attributable to the clinical characteristics of the sample, timing of trauma, and particularly the distinction between “*self-perceived*” growth and functional recovery. While Tedeschi and Calhoun emphasized the constructive and adaptive aspects of PTG, Zoellner and Maercker proposed a two-component model suggesting that PTG may also encompass self-deceptive or dysfunctional forms of “illusory growth” [66,67]. According to this model, perceived growth may serve as a short-term cognitive coping mechanism; however, it may coexist with ongoing psychopathology over time. In our sample, the relatively low levels of PTG in the MDD group, together with its lack of association with suicide risk, may indicate that persistent depressive symptoms are linked to reduced cognitive flexibility and meaning-making capacity, potentially attenuating the protective role of PTG. Furthermore, retrospective evaluations of CT may be influenced by psychopathology-related alterations in autobiographical memory, and the subjective meaning attributed to traumatic experiences may change over time and be shaped by subsequent life events [68], which can complicate the understanding of how these experiences impact current mental health outcomes.

The effects of protective factors may be context-, sample-, and time-specific; thus, their interpretation is not always linear or straightforward [46]. Particularly in clinical populations, the burden of acute symptoms may obscure or modify the influence of protective factors. Prospective longitudinal studies across different psychiatric disorders are therefore needed to clarify the complex and potentially bidirectional associations among PTG, mental disorders, and suicide risk.

MDD most commonly emerges during early adulthood, a period typically characterized by peak productivity, and is associated with significant functional impairment. Epidemiological evidence suggests that MDD is approximately twice as prevalent in women as in men, with an estimated heritability rate of around 37% [46,69]. In our sample, the higher rates of family history of psychiatric disorders and unemployment observed in the MDD group align with the sociodemographic characteristics frequently documented in the depression literature. Nevertheless, future studies that include broader age groups—such as adolescents and older adults—and allow for gender-based comparisons are required to further evaluate the generalizability of these findings.

Several limitations should be considered when evaluating the findings of this study. First, CT was assessed retrospectively through a self-report scale, which may introduce recall bias and may also be influenced by the participants’ current psychological state. Additionally, the chronicity, duration, and repetitive nature of trauma exposure were not thoroughly evaluated. Second, the cross-sectional design of the study prevents causal inferences about the directionality of the observed associations. Although mediation analyses were conducted to explore potential relational patterns among the variables, these findings should not be interpreted as evidence of causality. Third, the sample consisted of patients with MDD who were receiving antidepressant treatment, and therefore, the potential confounding effects of pharmacological treatment on symptom severity and psychological variables could not be fully controlled. Furthermore, the study was conducted in a single clinical center and included participants within a restricted age range, which may limit the generalizability of the findings to broader populations. Future studies employing longitudinal and multicenter designs, including drug-naive patient cohorts and more diverse age groups, and incorporating more detailed assessments of trauma characteristics may help clarify the temporal and potentially bidirectional relationships among the variables.

## 6. Conclusions

Our findings indicate a complex relationship between childhood trauma, dissociative experiences, suicide risk, and sleep quality in patients with MDD. In contrast, posttraumatic growth was not significantly associated with the clinical variables in this population. Dissociative experiences were strongly associated with both suicide risk and sleep quality, suggesting a potential role in the clinical presentation of MDD. From a clinical perspective, these findings highlight the importance of carefully assessing dissociative symptoms, which are often overlooked or misinterpreted. Clinicians should also consider therapeutic interventions that specifically target these symptoms, particularly in individuals with MDD and a history of childhood trauma. Overall, adopting a multidimensional approach that considers both risk and protective factors may support more individualized and sensitive treatment strategies and ultimately improve clinical outcomes in MDD.

## Figures and Tables

**Table 1:** Sociodemographic features of the participants.

	MDD (*n* = 115)	Control (*n* = 84)	*p*
**Age, m** **ean ± SD**	29.84 ± 7.67	31.44 ± 5.92	0.099
**Gender**			
Female	85 (73.9%)	62 (73.8%)	0.987
Male	30 (26.1%)	22 (26.2%)	
**Marital status**			
Single	68 (59.1%)	38 (45.2%)	0.052
Married	47 (40.9%)	46 (54.8%)	
**Occupational status**			
Unemployed	74 (64.3%)	10 (11.9%)	**<0.001**
Employed	41 (35.7%)	74 (88.1%)	
**Place of residence**			
Rural	7 (6.1%)	4 (4.8%)	0.686
Urban	108 (93.9%)	80 (95.2%)	
**Family history of mental disorders**			
No	73 (63.5%)	73 (86.9%)	**<0.001**
Yes	42 (36.5%)	11 (13.1%)	
**Smoking**			
No	69 (60%)	59 (70.2%)	0.136
Yes	46 (40%)	25 (29.8%)	

MDD: Major depressive disorder. *p* values in bold are statistically significant (*p* < 0.05).

**Table 2:** Comparison of the scale scores of the groups.

	MDD (*n* = 115)	Control (*n* = 84)	Effect Size	*p*
Hamilton Depression Rating Scale	12.90 ± 6.05	1.21 ± 1.91	2.45	<0.001 ^a^
Beck Scale for Suicidal Ideation	4.0 [0.0–32.0]	0.0 [0.0–0.0]	0.90	<0.001 ^b^
Posttraumatic Growth Inventory-total	43.52 ± 19.98	60.56 ± 21.78	0.82	<0.001 ^a^
Dissociative Experiences Scale	32.36 ± 21.77	11.61 ± 10.68	1.16	<0.001 ^a^
Pittsburgh Sleep Quality Index-total	9.91 ± 4.35	5.79 ± 3.22	1.05	<0.001 ^a^
Childhood Trauma Questionnaire-total	63.56 ± 19.60	41.87 ± 10.52	1.32	<0.001 ^a^
Emotional abuse	11.03 ± 5.22	6.50 ± 2.11	1.08	<0.001 ^a^
Emotional neglect	15.06 ± 4.92	8.68 ± 3.59	1.45	<0.001 ^a^
Physical abuse	6.0 [5.0–25.0]	5.0 [5.0–12.0]	0.60	<0.001 ^b^
Physical neglect	9.79 ± 3.78	6.93 ± 2.62	0.86	<0.001 ^a^
Sexual abuse	5.0 [5.0–25.0]	5.0 [5.0–11.0]	0.37	<0.001 ^b^
Overprotection-overcontrol	12.86 ± 4.72	9.11 ± 3.59	0.88	<0.001 ^a^

MDD: Major depressive disorder. ^a^ Independent samples *t*-test. ^b^ Mann–Whitney *U* test.

**Table 3:** The correlations between scale scores in the MDD group.

	BSSI	DES	CTQ Total	Emotional Neglect	Emotional Abuse	Physical Neglect	Physical Abuse	Sexual Abuse	OP-OC	PSQI	PTGI
HDRS	0.372 **	0.323 **	0.199 *	0.215 *	0.147	0.281 **	0.190 *	0.119	0.065	0.368 **	−0.342 **
BSSI	1.000	0.381 **	0.080	0.006	0.083	0.138	0.099	0.193 *	−0.050	0.265 **	−0.110
DES			0.313 **	0.354 **	0.356 **	0.256 **	0.220 *	0.096	0.123	0.386 **	−0.117
CTQ total			1.000	0.830 **	0.824 **	0.630 **	0.762 **	0.330 **	0.691 **	0.297 **	−0.059
Emotional Neglect				1.000	0.658 **	0.611 **	0.597 **	0.183	0.504 **	0.333 **	−0.106
Emotional Abuse					1.000	0.490 **	0.575 **	0.261 **	0.567 **	0.270 **	−0.018
Physical Neglect						1.000	0.517 **	0.187 *	0.213 *	0.241 **	−0.163
Physical Abuse							1.000	0.244 **	0.418 **	0.296 **	−0.113
Sexual Abuse								1.000	0.079	0.114	−0.015
OP-OC									1.000	0.132	−0.008
PSQI										1.000	−0.134

HDRS: Hamilton Depression Rating Scale BSSI: Beck Scale for Suicidal Ideation, DES: Dissociative Experiences, Scale CTQ: Childhood Trauma Questionnaire, OP-OC: Overprotection-overcontrol, PSQI: Pittsburgh Sleep Quality Index, PTGI: Posttraumatic Growth Inventory; *p* < 0.05 = *, *p* < 0.001 = **.

**Table 4:** Comparison of scale scores between MDD patients with and without suicide attempts.

	Suicide Attempts (–) (*n* = 84)	Suicide Attempts (+) (*n* = 31)	Effect Size	*p*
Hamilton Depression Rating Scale	12.57 ± 5.94	13.77 ± 6.34	0.20	0.346 ^a^
Beck Scale for Suicidal Ideation	3.0 [0.0–22.0]	13.0 [1.0–32.0]	0.50	**<0.001 ^b^**
Posttraumatic Growth Inventory-total	42.83 ± 19.88	45.39 ± 20.45	0.13	0.545 ^a^
Dissociative Experiences Scale	29.45 ± 19.30	40.25 ± 26.11	0.51	**0.042 ^a^**
Pittsburgh Sleep Quality Index-total	9.62 ± 4.65	10.71 ± 3.31	0.25	0.167 ^a^
Childhood Trauma Questionnaire-total	60.89 ± 18.48	70.77 ± 21.02	0.51	**0.016 ^a^**
Emotional abuse	10.38 ± 4.92	12.25 ± 5.41	0.37	0.064 ^a^
Emotional neglect	14.22 ± 4.60	16.00 ± 5.04	0.38	0.104 ^a^
Physical abuse	6.0 [5.0–25.0]	8.0 [5.0–22.0]	0.21	**0.021 ^b^**
Physical neglect	9.44 ± 3.68	10.25 ± 3.89	0.22	0.526 ^a^
Sexual abuse	5.0 [5.0–23.0]	5.0 [5.0–25.0]	0.23	**0.011 ^b^**
Overprotection-overcontrol	12.11 ± 4.31	13.75 ± 4.98	0.36	0.183 ^a^

^a^ Independent samples *t*-test. ^b^. Mann–Whitney *U* test. *p* values in bold are statistically significant (*p* < 0.05).

**Table 5:** The mediating role of dissociative experiences in the relationship between depression severity and suicidal ideation.

Model	Pathway	Coefficient *(β)*	Standard Error	*p*
l. Basic model (without M)	*X → Y*	0.426	0.108	<0.001
ll. Mediation analysis	*X → M*	1.103	0.322	<0.001
ll. Mediation analysis	*M → Y*	0.108	0.030	<0.001
ll. Mediation analysis (direct effect)	*X → Y*	0.306	0.108	0.005
ll. Mediation analysis (indirect effect)	Pathway	Coefficient *(β)*	Standard Error	Bootstrap Confidence interval BootLower limit/BootUpper limit
	*X → M → Y*	0.120	0.046	0.043/0.223

**Table 6:** The mediating role of dissociative experiences in the relationship between childhood traumas and sleep quality.

Model	Pathway	Coefficient *(β)*	Standard Error	*p*
l. Basic model (without M)	*X → Y*	0.066	0.020	0.001
ll. Mediation analysis	*X → M*	0.347	0.099	<0.001
ll. Mediation analysis	*M → Y*	0.064	0.018	<0.001
ll. Mediation analysis (direct effect)	*X → Y*	0.043	0.020	0.032
ll. Mediation analysis (indirect effect)	Pathway	Coefficient *(β)*	Standard Error	Bootstrap Confidence interval BootLower limit/BootUpper limit
	*X → M → Y*	0.022	0.009	0.006/0.043

## Data Availability

The data presented in this study are available on request from the corresponding author due to the presence of sensitive clinical and personal information.
